# Clinical characteristics and comorbidities of elderly asthmatics who attend allergy clinics

**DOI:** 10.1186/s40733-018-0041-x

**Published:** 2018-04-23

**Authors:** Anahí Yáñez, Marcela Soria, Susana De Barayazarra, Nancy Recuero, Francisco Rovira, Edgardo Jares, Ana María Stok, Sergio Nemirovsky, Carlos Bueno

**Affiliations:** 1 Investigaciones en Alergia y Enfermedades Respiratorias (INAER), Buenos Aires, Argentina; 2 Hospital Zona General de Agudos Dr. Ricardo Gutiérrez, La Plata, Argentina; 3 Allergy and Immunology Service, Hospital San Roque, Córdoba, Argentina; 4 Argentine Respiratory Medicine Association, Buenos Aires, Argentina; 5 Private Medical Centers SA, Ramos Mejía, Buenos Aires Argentina; 6 Instituto de Investigaciones en Patologías Respiratorias, San Miguel de Tucumán, Tucumán Argentina; 7 CONICET - Universidad de Buenos Aires. Instituto de Química Biológica de la Facultad de Ciencias Exactas y Naturales (IQUIBICEN), Buenos Aires, Argentina; 8 Facultad de Ciencias Exactas y Naturales, Departamento de Química Biológica, Universidad de Buenos Aires, Laboratorio de Virología, Buenos Aires, Argentina

**Keywords:** Asthma, Elderly, Elderly, Comorbidities, Characteristics, Allergy

## Abstract

**Background:**

To date, few studies have focused on the clinical and allergic characteristics of asthma in the elderly, defined as asthma in people aged 60 or over. Thus, we propose to identify and study the clinical and allergic characteristics and comorbidities of patients with asthma among the elderly.

**Methods:**

A retrospective, observational, descriptive study was developed in five clinics and hospitals in Argentina. Allergy Physicians analyzed their patients’ medical records in 2014 and included those adults over the age of 60, who had been diagnosed with asthma according to the GINA guidelines. Clinical and allergic characteristics were analyzed.

**Results:**

A total of 152 patients diagnosed with asthma, of whom 73% were women and 11% ex-smokers, were included in this study, with a mean age of 66 years. Only 10.5% of the participants had onset asthma past the age of 60. Regarding asthma severity, 74.3% were diagnosed with moderate persistent asthma, and 7.2% with severe persistent asthma. Eighty-four percent of the patients were treated with an inhaled corticosteroid (ICS) along with a long-acting β 2-adrenergic agent (LABA). More than half of the patients had two or more comorbidities simultaneously. Allergic comorbidities were the most frequent comorbidities, followed by arterial hypertension. Among allergic comorbidities, most patients presented allergies at the nasal level. There were no significant differences between the subpopulations of patients with late-onset asthma (LOA) and asthma with onset before the age of 60, i.e. early onset asthma (EOA) in most of their clinical characteristics. However, it was observed that EOA accounted for a higher percentage of patients with nasal allergies as compared to LOA (71% vs 46%, *p* <  0.05).It is worth mentioning that almost half of the patients with LOA had allergies at the nasal level.

**Conclusion:**

These results may provide a better understanding of the clinical characteristics of asthma in the elderly in Argentina, thus, enabling the development of future therapeutic strategies and a better quality of life for our elderly asthma patients.

## Background

In the past, asthma was mainly considered a childhood illness. However, recent epidemiological studies indicate that new incident asthma can occur at any age, and it is in fact highly prevalent in the older adult population and its prevalence ranges from 4.5% to 12.7%. According to the current demographic trends, a 100% increase in the number of people over the age of 65 is expected in the next 20 years [1, 2]. It would therefore be reasonable to assume that there will be an increase in the impact of asthma on the elderly in the near future. Likewise, the burden of asthma is significantly higher in the elderly than in the young, particularly in relation to mortality, hospitalization, medical costs, and quality of life. However, asthma in the elderly is often underdiagnosed and undertreated [1, 2].

The mainstay of asthma management at any age is to achieve and keep the disease under control. The pharmacological control depends on the clinical and allergic characteristics of the disease, and, in addition, symptoms may be influenced by age-related factors such as an increased frequency of comorbidities and patients prescribed with multiple drugs simultaneously. In addition, specific problems associated with the management of asthma in the elderly have been described, such as improper use of the inhaler, poor adherence to treatment, increased adverse effects, and declining response to medications. The study of the clinical characteristics of asthma in the elderly is of utmost importance in terms of providing evidence to identify those diagnostic and therapeutic methods that are safe and effective [1, 3].

Comorbidities are considered to be some of the key characteristics of geriatric patients and contribute to the clinical complexity of the elderly. In addition, they contribute to poor asthma management outcomes in this group as compared to younger asthmatic patients. The presence of comorbidities has been shown to influence the quality of life of the elderly with asthma, which in turn affects adherence to treatments. In this context, specific comorbidities may also prevent the correct use of inhaler devices, for example, in patients suffering from arthritis, orthodontics, dementia, etc [3, 4].

Knowing the epidemiology of asthma is essential for the planning of health services, but our knowledge about this disease is still incomplete. Historically, clinical studies on asthma have frequently excluded the elderly population, and there are still few observational epidemiological studies which could shed light on the nature or the pathophysiology of the disease. Consequently, it is imperative to study the clinical and allergic characteristics of asthma in the elderly, on the one hand, and their comorbidities, on the other hand, in order to propose new therapeutic and diagnostic strategies, and ultimately, to improve the patients’ quality of life [1, 5].

Therefore, we aim to identify and study the clinical and allergic characteristics, as well as the medical and allergic comorbidities of asthma in patients over the age of 60 in Argentina.

## Methods

A retrospective, observational, descriptive study was developed in five clinics and hospitals in Argentina: Allergy and Respiratory Diseases Research (InAER), Autonomous City of Buenos Aires, Argentina; Hospital Area General De Agudos Dr. Ricardo Gutierrez, La Plata, Argentina; Allergy and Immunology Service, Hospital San Roque, Cordoba, Argentina; Private Medical Centers, Ramos Mejia, Buenos Aires, Argentina. Allergists analyzed their patients’ medical records for three months in 2014, and included those aged 60 or older diagnosed with asthma, according to the GINA guidelines. It should be noted that some patients did not present reversibility in the cross-section of this study, although prior to the diagnosis of asthma they did present it, according to the GINA guidelines.

For each asthmatic patient over 60 years, researchers gathered the following information: 1) age, sex and race; 2) smoking status; 3) age at the onset of asthma, defined as the age at which the patient is diagnosed with asthma; 4) asthma severity (according to the GINA guidelines); 6) Pulmonary function values; 7) positive reaction to the Skin Prick Test (SPT) (mites, cats, dogs, aeroallergens, pollen); 8) exacerbation triggers (exercise, tobacco smoke, occupational, NSAIDs, menstruation, allergens); 9) total serum IgE (IgE ˃ 100 UI/ml were considered subjects with high IgE); 10) eosinophil count (eosinophil count in the peripheral blood of ≥500 eosinophils/μl were considered eosinophilic subjects),; 11) current treatment they receive; 12) dose of inhaled corticosteroids; 13) Medical comorbidities (hypertension, gastroesophageal reflux (GERD), dyslipidemia, coronary heart disease, diabetes, pulmonary hypertension, other cardiovascular diseases) and allergic conditions (seasonal allergic rhinitis, perennial allergic rhinitis, allergic conjunctivitis, sinusitis, chronic rhinosinusitis, nasal polyposis, eczema, chronic urticaria, acute urticaria, intolerance to non-steroidal anti-inflammatory drugs (NSAIDs), eosinophilic esophagitis).

### Statistical analysis

Data are expressed as a percentage, except for the age of onset and the time span with asthma, which are presented as mean and standard deviation (SD). The clinical characteristics of the EOA and LOA subpopulations were compared using unpaired Student *t* and Fisher’s Exact Test, as appropriate. *P* values ​​below 0.05 were considered to be statistically significant.

## Results

A total of 152 patients with a defined diagnosis of asthma were included in this study, with a mean age of 66 (SD 6.5) years, of whom the majority were women (73%). Regarding the racial characteristics of the participants, all of them were were Caucasian. None of the patients were currently smokers, and only 11% had previously smoked. The mean age of onset of asthma symptoms was 33.4 (SD 22.9) years, with an average of 34.2 years (SD 21.9) (Table 1).

**Table 1:** Demographic, clinical and functional data of the study subjects

	All subjects	LOA	EOA	*p*
Subjects, %	100 (*n* = 152)	10.5 (*n* = 16)	89.4 (*n* = 136)	
Female, %	73%	74%	68%	ns
Age, yrs	66 (SD 6.5)	76 (SD 7)	65 (SD 5)	< 0.0001
Former Smokers	11%	6,2%	14,2%	ns
Age of onset, yrs	33.4 (SD 22.9)	69.2 (SD 6.8)	29 (SD 12.4)	< 0.0001
Asthma duration, yrs	34.2 (SD 21.9)	7.3 (SD 4)	37.4 (SD 21)	< 0.0001
Asthma severity				ns
severe persistent,%	7.2	13	7	ns
moderate persistent,%	74.3	75	74	ns
mild persistent,%	15.7	12	16	ns
Intermittent,%	2.6	0	3	ns
FEV_1_^1,^ %	73 (SD 21)	71 (SD 20)	73 (SD 21)	ns
FVC/FEV_1_^2^, %	69 (SD 12)	68 (SD 11)	70 (SD 12)	ns
Subjects with normal FEV_1_, %	39	50	37	ns
Subjects with reversibility, %	59	62	58	ns
Subjects with high IgE, %	54	72	51	ns
IgE (UI/ml)	181 (SD 68)	183 (SD 70)	177 (SD 75)	ns
Eosinophilic subjects, %	52	50	54	ns
Eosinophil count, %	6,1	6,3	4,3	ns

Data are expressed as mean ± SD if not otherwise stated

Late-onset asthma (LOA), defined as the onset of asthma after the age of 60, was observed in 10.5% of the patients, with a mean age of onset of 69.2 (SD 6.8) years, having had asthma for an average of 7.3 (SD 4) years. On the other hand, the mean age of onset of symptoms in patients with asthma with an onset before the age of 60 (EOA) was 29 (SD 12.4) years, having had the disease for 37.4 (21 years); both the age of onset of asthma and the time span with asthma were significantly different between both subpopulations (*p* <  0.0001) (Table 1).

With regard to the severity of asthma, 74.3% of the patients were diagnosed with moderate persistent asthma, 15.7% with mild persistent asthma, 7.2% presented with severe persistent asthma, and 2.6% with intermittent asthma, with no statistical difference between the EOA and LOA populations (Table 1).

The mean FEV_1_ (forced expiratory volume) was 73%, and FEV_1_/forced vital capacity (FVC) was 69%. In addition, 39% presented normal values ​​of FEV_1_ (higher than 80%), with 59% reversibility. Among the LOA patients, we found that 50% had normal FEV_1_ (mean FEV_1_ of 71%), and 62% were reversible. While among the EOA patients, 37% had normal FEV_1_ (mean FEV_1_ of 73%), and 58% were reversible, with no statistical difference between the EOA and LOA populations (Table 1).

Of all the patients tested on the SPT (72% of the subjects), only 4% did not present any positive response. Among the allergens investigated, mites (70%), fungi (58%) and pollen (32%) were included (Table 2).

**Table 2:** Comparison of allergens between the total population and LOA and OAE

	All subjects	LOA	EOA	*P*
Allergens				
Mites, %	70	50	70	ns
Fungi, %	58	100	56	ns
Pollen, %	32	50	31	ns
None, %	4	0	4	ns

Among the asthma exacerbation triggers reported by the patients, tobacco smoke was predominantly found, resulting from passive smoking (75%), exercise (39%) and aeroallergens (21%). Also, 31% of the patients presented other triggers not included among the mentioned ones, such as dyes, perfumes and temperature changes. On the other hand, 4% of the patients did not present any triggers (Table 3).

**Table 3:** Comparison of Asthma exacerbation triggers between total population and LOA and EOA

	All subjects	LOA	EOA	*P*
Triggers				
Tobacco smoke, %	75	63	73	ns
Aeroallergens, %	21	6	22	ns
Exercise, %	39	31	40	ns
Others, %	31	19	34	ns
None, %	4	13	3	ns

Among the EOA and LOA populations, there were no significant differences in both positive SPT allergens and exacerbation triggers (Tables 2 and 3).

IgE was elevated in 54% of the patients, with an average of 181 IU/ml, and eosinophilia was detected in 53% of the patients, with an average of 6.1% of eosinophils in the blood, with no significant differences among the EOA and LOA patients (Table 1).

### Asthma treatment

Eighty-three percent of the patients were treated with an inhaled corticosteroid (ICS) along with a long-acting β-adrenergic agent. Sixty percent of all patients used fluticasone and salmeterol, while 24% used budesonide and formoterol. The ICS dose was greater than 1000 mg/day in 24% of the patients. On the other hand, 16% had not been prescribed any specific treatment for asthma. The main rescue treatment option chosen by the patients was fast-acting β-adrenergic agonists, with salbutamol being the most widely used one (91%), and only a few of the patients used ipratropium bromide. On the other hand, 9% of the patients did not use any rescue treatment. Among the EOA and LOA patients, no significant differences were observed in terms of the treatments used (Table 4).

**Table 4:** Comparison of treatments between the total population and LOA and OAE

	All subjects	LOA	EOA	*P*
Rescue treatment				
Salbutamol, %	91	88	91	ns
Ipratropium bromide, %	1	0	1	ns
None, %	9	12	9	ns
Base treatment				
Fluticasone and salmeterol, %	60	63	60	ns
Budesonide and formoterol, %	24	25	24	ns
None, %	16	12	17	ns
ICS dose greater than 1000 mg/day, %	24	25	23	ns

### Comorbidities

Thirty-six percent of the patients experienced three or more comorbidities simultaneously, two comorbidities were observed in 32%, one comorbidity in 26%, and only 6% of the patients did not present any comorbidities at all. Allergic comorbidities were most frequently found (65%), followed by hypertension (45%), GERD (16%), diabetes (12%), obesity (9%), coronary diseases (8%), osteoarthritis, arthritis (7%), hypothyroidism (7%), Chagas disease (4%), and dyslipidemia (3%). Other comorbidities were also found, such as gastritis, celiac disease, breast cancer and pulmonary hypertension, but only in a few patients. There were no significant differences in the percentages of these comorbidities between the EOA and LOA patients (Table 5).

**Table 5:** Comparison of comorbidities between the total population and LOA and OAE

	All subjects	LOA	EOA	*p*
Comorbidities				
None, %	6	13	6	ns
3 or more, %	36	25	36	ns
2, %	32	31	32	ns
1, %	26	31	26	ns
Hypertension, %	45	50	45	ns
Allergic comorbidities, %	65	50	67	ns
GERD, %	16	0	18	ns
Obesity, %	9	6	10	ns
Diabetes, %	12	0	12	ns
Osteoarthritis, %	7	6	7	ns
Hypothyroidism, %	7	6	7	ns
Chagas disease, %	4	0	4	ns
Coronary diseases, %	8	6	8	ns
Dyslipidemia, %	3	0	4	ns

Among allergic comorbidities, patients mainly had nasal allergies (68%). Among them, mainly perennial rhinitis (23%), seasonal (18%) or chronic rhinosinusitis (15%), and, in addition, some patients presented chronic rhinosinusitis and perennial rhinitis simultaneously (12%). Only a few patients had chronic rhinosinusitis and seasonal rhinitis (1%). Other allergic comorbidities were uncommon, such as allergic conjunctivitis, sinusitis, nasal polyposis, eczema, chronic or acute urticaria, and intolerance to NSAIDs. Twenty-nine percent of the patients did not present any of the comorbidities sought. On the other hand, EOA patients represented a higher percentage of patients with nasal allergies as compared to LOA (71% vs 46%, *p* <  0.05). However, it is worth noting that almost half of the patients with LOA had chronic rhinosinusitis or allergic rhinitis. Among the patients with EOA, we observed that a similar proportion of patients had seasonal rhinitis, perennial rhinitis and chronic rhinosinusitis (17, 23 and 15%, respectively), while 13% had chronic rhinosinusitis and perennial rhinitis. Only a small percentage of patients had chronic rhinosinusitis and seasonal rhinitis [1]. Likewise, there were no significant differences in the proportions of patients with seasonal rhinitis, perennial and chronic rhinosinusitis, between the EOA and LOA populations (Table 6).

**Table 6:** Comparison of nasal allergies between the total population and LOA and OAE

	All subjects	LOA	EOA	*P*
Nasal allergies	68	46	71	< 0.05
Chronic rhinosinusitis, %	15	7	15	ns
Seasonal rhinitis, %	18	20	17	ns
Perennial rhinitis, %	23	20	23	ns
Chronic rhinosinusitis and Perennial rhinitis, %	12	0	13	ns
Chronic rhinosinusitis and Seasonal rhinitis, %	1	0	1	ns

## Discussion

Asthma in the elderly is a health problem that, despite being frequent, ends up being both under identified and under diagnosed, which leads to inconveniences in the patient’s quality of life, and associated morbidity and mortality [1, 2]. The results obtained from this population demonstrate different phenotypic differences with respect to that of young patients, which has potential impact on the diagnosis and management of this population. The chances of multi-causality of symptoms or physical dysfunctions are much more likely at this age. The response to medications is also more difficult to interpret. Consequently, a greater knowledge of its epidemiology, its clinical characteristics, comorbidities and treatment is indispensable [1, 2].

In this paper, we have been able to identify for the first time the clinical characteristics and comorbidities of elderly asthmatics in Argentina. To the best of our knowledge, this is the first time that a study like this one has been done in Argentina, as well as in Latin America.

As in other studies conducted in Italy, Japan and the United States [5–8], in this study, women are more represented than men, which is possibly due to a higher prevalence of women in the larger populations owing to their greater longevity, or a greater diagnosis of COPD in men, or other factors. Given the importance of smoking in the pathophysiology of respiratory diseases, it is important to note that there were no current smokers in this population, and only 11% had previously smoked.

For many years asthma in the older adult had been considered non-atopic [9], but recent studies show that it is also atopic, with percentages of allergies ranging from 30 to 77% [7, 8, 10, 11]. In addition, a recent study performed in the United States with a large population of patients, reported a percentage of allergies of approximately 65% ​​in elderly asthmatics, which is similar to that observed in young asthmatic patients. In this sense, in the population described in this study, only 4% of the patients did not present any positive response to the SPT. However, given that all patients were selected from allergy practices, we cannot rule out the possibility of selection bias It is important to note that 7% of this population had positive SPT and severe persistent asthma, being potential candidates for anti-IgE therapy. In previous studies, similar percentages of adult asthmatic populations could potentially be treated with this therapy. Given these results, and as suggested by Scichilone et al., the study of sensitivity to allergens in elderly asthma patients could be recommended [12].

It is noteworthy that the mean values ​​of IgE in this population (181 IU/ml) and eosinophils (6.1%) were higher than those reported in the study by Inoue et al. (91 IU/ML and 0.5%, respectively) [5]. The eosinophil count was also higher than that reported by Park et al. [11]. One possible explanation may be that the patients included in this study were mostly atopic, whereas in the work of Inoue et al. and Park et al. they represent approximately half of them [5, 11].

On the other hand, the values ​​obtained from FEV_1_ (73%), and FEV_1_/FVC (69%) were similar to those obtained by Park et al. and Inoue et al., who reported values ​​of 75% and 66%, and 81% and 72%, for FEV_1_ and FEV_1_/FVC, respectively. In both studies it was observed that values ​​are significantly lower than when compared to non-elderly asthmatics. It is important to emphasize that the population described in this study presents an average age lower than the average age of the patients described in the studies of Inoue et al. and Park et al. (66 years vs 73 years and 70 years, respectively), and the duration of the illness is much longer (34.2 years vs 12.7 years and 11.8 years, respectively). In this sense, Inoue et al. demonstrated that asthma in the older adult is characterized by an airway obstruction that is independent of age per se and the duration of the disease [5, 11].

As with other populations of elderly asthma patients, most patients are treated with ICS along with a long-acting β-adrenergic agent [1, 6, 7]. Although in this population, 16% of the patients do not have any specific treatment prescribed for asthma, in other studies it has also been observed that approximately 10% do not receive any treatment [1, 6, 7].

Most patients presented comorbidities, as described for older asthmatic adults [1]. However, unlike Milanese et al. [6], one (26%), two (32%), or three or more comorbidities (36%) were found to be similar in the population described here, with almost half of the patients presenting with two comorbidities.

However, the comorbidities found match what has been previously reported for this population with a high frequency of arterial hypertension and allergic comorbidities, and, to a lesser extent, diabetes mellitus, cardiovascular diseases, and osteoarthritis or arthritis [1, 6, 7]. Older patients with asthma are usually divided into two groups: those who have had asthma since childhood (EOA) and those who develop it in adulthood (LOA) [9, 13, 14]. In our study, we divided them into those who acquired asthma after the age of 60 (LOA) [14], and those who acquired it before the age of 60 (EOA) in a manner similar to that described by Battaglia et al. and Braman et al. [9, 14]. Considering the previously described association between allergic rhinitis and EOA, in this population, the majority of EOA patients presented with allergic rhinitis or chronic rhinosinusitis (71%), with statistically significant differences with respect to the LOA population (46%). However, we also observed that almost half of the patients with LOA presented with chronic rhinosinusitis or allergic rhinitis [1, 15] without significant differences in the proportions of patients with seasonal rhinitis, perennial and chronic rhinosinusitis between both subpopulations. This may be associated with an increasing problem of allergic rhinitis in the older adult population. In an epidemiological study of atopic asthma, the high prevalence of allergic rhinitis and atopic dermatitis observed in the young population with allergy was similar in the elderly population [16]. Similarly, the prevalence of allergic rhinitis among people aged 60-70 years in Switzerland is around 13-15% [1]. On the other hand, it has been described that subjects with EOA have a greater irreversible airway obstruction compared to those with LOA [5, 13, 17]. However, in this study we obtained statistically similar values ​​of FEV1, FEV1/FVC for both populations, and, in addition, the percentage of patients with normal FEV1 and reversibility was also similar. It is important to note that in this study the proportion of subjects with LOA was much lower as compared to EOA. Moreover, in two studies comparing EOA vs LOA, the FEV1 values ​​ obtained for the LOA population are similar to those observed in this study, while the values ​​corresponding to EOA subjects are much lower than in this study, which in comparison are significantly different [9, 17]. In this regard, it is worth noting that in many of the studies, EOA patients present the onset of asthma during childhood, whereas in this study the average age of onset of asthma in the EOA population was 29 years-old. Besides, in one of these studies, both populations were discriminated on the basis of the time they experienced asthma (the cut-off limit is 26 years with asthma) rather than at the onset age of asthma [17]. Our study has a series of advantages, such as the large sample size, the uniformity in the gathering of information, and the staging according to international asthma recommendations. However, some limitations may also be listed. In our study there was a predominance of women and Caucasian population, which limits the generalization of the results to other populations. Besides, the numbers of subjects are especially small (*n* = 16) in the LOA group.

Recruitment was in Allergy Units, although many asthmatics are also treated by primary care Physicians, Pulmonologists and internists. Also, not all the population had skin tests performed and/or diagnosis and treatment of upper airway punctuated. On the other hand, some of these patients may also display an ASTHMA-COPD overlap syndrome [18]. Finally, since the study design is transversal, we cannot infer causality in the reported associations. In spite of this we can conclude that we have found a greater proportion of the elderly population with atopic asthma and with more comorbidities than in other developed countries. Likewise, the EOA and LOA patients included in this study did not show significant differences in most of their clinical and allergic characteristics studied. However, there were differences in allergies at the nasal level, which made it possible to identify the association between allergic rhinitis and EOA previously described [1].

## Conclusions

These results may provide a better understanding of the clinical characteristics of asthma in the elderly, and thus enable the development of future therapeutic strategies and a better quality of life for our elderly asthmatic patients.
